# The Interaction between Message Sensation Value and Food Neophobia in Communication about Insect-Based Foods: An Experiment with Italian Consumers

**DOI:** 10.3390/nu15010191

**Published:** 2022-12-30

**Authors:** Roberta Riverso, Mario Amato, Fabio Verneau, Francesco La Barbera

**Affiliations:** Department of Political Science, University of Naples Federico II, Via Rodinò 22/A, 80138 Naples, Italy

**Keywords:** consumer behavior, novel foods, *Acheta domesticus*, food sustainability

## Abstract

Currently, insects are considered as a promising alternative protein source due to their nutritional content and their environmental sustainability. Notwithstanding this, generally consumers show reluctance towards the introduction of edible insects into their diet, mostly influenced by food neophobia. Persuasive communication strategies (e.g., informational vs. emotional appeals) have been a major topic in consumer behavior research. Scholars often refer to the construct of message sensation value (MSV), which is defined as the intensity of audio, visual, and content features of a message that elicit sensory, affective, and arousal responses. In this work, a computer-based experiment (N = 148) was conducted to investigate the effectiveness of messages based on different levels of MSV in promoting the intention to eat insect-based foods, and interactions between MSV and food neophobia. Results indicate that, MSV, food neophobia, and their interaction significantly affect the willingness to consume insect-based food products with or without visible insects, highlighting novel pathways for segmenting consumers, in order to strengthen the communication effects.

## 1. Introduction

In recent years, there has been a growing interest towards edible insects, both from academia and stakeholders. The main thrust that has led insects to be elected as a viable alternative protein source is the need to meet the growing energy and protein needs that future generations will face. Indeed, the global population will reach almost 10 billion by 2050 [1] and, subsequently, animal proteins availability will need to increase to more than 70% in order to satisfy the demand [2]. Insects’ nutrient composition can meet, or even exceed, other traditionally consumed animals or plants [3]. Furthermore, the natural presence of chitin may represent a good source of fiber and might enhance the immune system [3]. Beside the rich nutritional composition, it is important to highlight that insects have a more efficient feed-conversion rate [4], lesser emissions of greenhouse-gasses and ammonia, and lesser land use and water consumption if compared to conventional animal proteins [5,6]. Nowadays, in Europe, edible insect products fall under the scope of Regulation (EU) 2015/2283 and, therefore, can be used as main ingredient in novel foods or as an ingredient of well-known foods destined for human consumption if a prior marketing authorization, issued by the EU Commission, has been obtained. Unlike the past, the inclusion of a product in the novel foods list has a broad and generic effect that permits other business operators to enter the market with an authorized novel food product without submitting another specific application. This openness had a positive impact upon edible insects food business operators that placed 500 tons of insect based products on the market in 2019 [7], forecasting this to be more than 250,000 tons by 2030. The forecast of a potential shift in product placement is mainly driven by consumer acceptance and change in Westerners’ sociocultural aspects, such as the increasing demand for meat analogues/alternatives. Nevertheless, Westerners often showed reluctance towards accepting edible insects as part of their diet [8] and broad scholarly consensus exists about unfamiliarity being a primary reason for low acceptance, jointly with food neophobia (the fear of trying new and unfamiliar food) and disgust [9]. Consumers’ willingness to eat edible insects increases when insects are processed and are thus undistinguishable in familiar food products, such as bread, biscuits, sauces, and soup [9,10,11,12,13,14,15].

### 1.1. The Role of Communication in Guiding Consumers’ Choice

Changing food preferences through persuasive communication has been one major topic of consumer behavior research [16,17,18]. Nevertheless, few studies have addressed the issue regarding insect-based food. A notable exception is represented by one experiment by Verneau and colleagues [18], in which different communication messages were compared. In this research, participants were invited to watch a video; the topic was manipulated on a random basis: societal benefits of eating insects and benefits of introducing tablets in schools (control group). Results showed that both messages, but especially the one based on societal benefits of the eating of insects, positively affected intention to eat insect-based foods, and eating behavior as well. It is easy to see how this work addresses the question of communication topics, without considering the issues related to the communication intensity. Overall, there is a lack of research on the intensity of messages in communication on direct and indirect entomophagy, especially in relation to the role of emotional appeals in communication. This is somewhat surprising, considering that in the scientific tradition of research on communication, one of the most common approaches has been to evoke emotions for increasing message effectiveness [19].

Persuasive communication consists in the use of messages aiming to change individuals’ beliefs and attitudes, in order to eventually obtain a modification of choices and behavior; this is commonly pursued through emotional and/or informational appeals [20]. In the realm of emotional appeals in persuasive communication, research often refers to the construct of message sensation value (MSV), which is defined as the intensity of audio, visual, and content features of a message that elicit sensory, affective, and arousal responses [21,22]. In other terms, MSV is an attribute of a message related to its content and formal features. Perceived message sensation value (PMSV), instead, is the degree to which the features of a message elicit sensory, affective, and arousal responses in subjects who receive the message [22]. Therefore, it is advisable to use the concept of MSV when making reference to the structural characteristics of the communication messages, and to use the concept of PMSV when studying and measuring affective, neuro-vegetative, physiological, and other kinds of responses to the communication messages. In a study based on participants’ self-reports, Palmgreen and colleagues [22] identified three dimensions of perceived MSV:

- emotional arousal: a message with high perceived MSV is seen as emotional, arousing, involving, exciting, and powerful;
- dramatic impact: a message with high perceived MSV is seen as dramatic, creative, and intense;
- novelty: a message with high perceived MSV is seen as novel and unusual.

**Figure 1:**
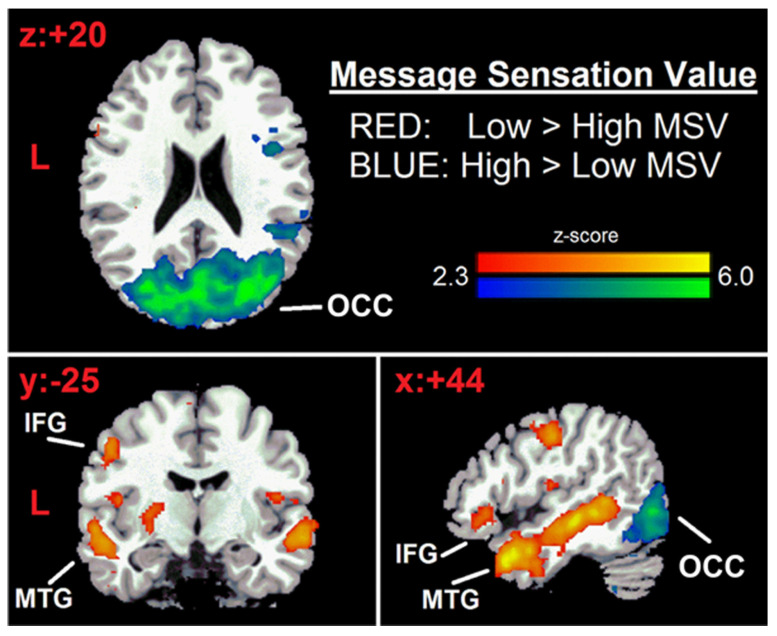
Brain activation in response to different MSVs. Source: [27].

MSV has been shown to affect communication impact and outcomes, and to play a major role in preferences for and reactions to persuasive messages [23,24,25,26]. The effects of messages with different MSVs have been clearly shown by fMRI data [27], highlighting that low vs. high MSV activates different brain areas (see Figure 1).

However, the impact of MSV on individuals is not that easily measured as it may appear, and the message intensity does not follow the rule of “the higher, the better”. Indeed, scholars have underlined relevant differences in the effects of high versus low MSV messages. High MSV messages are often used in commercial advertising and are thought to lead to an increased efficacy of persuasive communication [27,28]. This strategy received some empirical support, and several scholars propose that higher MSV brings increased attention and cognitive processing, leading to higher communication impact [23,29,30,31,32]. Nevertheless, experimental studies suggest that high MSV messages may result in reduced processing of content, reduced overall effectiveness, and that they do not always foster behavioral changes [28,33,34]. These negative results of high MSV messages have been explained in relation to their highly attention-demanding features, which may compete with the message content for individuals’ limited cognitive resources, thus reducing (rather than enhancing) the processing of message content. By contrast, communications characterized by low MSV have been found to be better remembered [27]. Therefore, it is important to study the effects of MSV in relation to different communication subjects and aims. Noticeably, the effectiveness of messages based on different levels of MSV in promoting intention to eat insect-based food has never been investigated.

In addition, MSV has been shown to interact with individual psychological factors such as sensation seeking and psychological reactance [24,35,36]. In the case of entomophagy, food neophobia (i.e., the tendency to avoid unfamiliar food) is the individual psychological factor most frequently cited and investigated to explain the aversion that consumers show for insects as food [18,37,38,39,40,41,42,43]. In order to measure this individual characteristic, Pliner and Hobden [44] validated the Food Neophobia Scale (FNS), which examines the human neophobia/neophilia continuum. As many scholars highlighted, food neophobia significantly and negatively affects individuals’ willingness to eat insect-based food [41,45,46,47]. Hitherto, the potential interactions between MSV and food neophobia in affecting the communication effectiveness on intention to eat insect-based foods have never been investigated.

The experiment described in the following sections was conducted in Italy with Italian participants. The Italian food culture has often been described as traditional [18], and edible insects have not yet gained widespread popularity in Italy; nevertheless, insect-based foods can be found in some very traditional Italian foods (such as Casu Marzu cheese), and the market for these alternative protein sources is slowly starting to grow [48]. In addition, some restaurants and specialty food stores in Italy have begun offering dishes and snacks made with insects, such as cricket pasta and mealworm crostini. In addition, there are a few Italian companies that produce and sell insect-based products, such as flour made from ground crickets or grasshoppers [48]. In sum, how quickly the edible insect market will grow in Italy remains an open question, but it is clear that there is an increasing interest in this sustainable and nutritious food source. Hence, the implementation of effective communication strategies may have a relevant impact over this ongoing process. Furthermore, previous research with Italian participants has shown significant difference as regards attitudes and intentions towards raw and processed insects, and food neophobia as well. Therefore, in order to answer the research questions, an Italian sample was considered appropriate.

### 1.2. Aims and Hypothesis

Our aim was to test the effectiveness of messages with different MSV in promoting intention to eat insect-based foods. Hence, we compared the effects of two messages (high MSV vs. low MSV) in influencing participants’ intention to eat foods containing—visible and non-visible—insects. In addition, we tested the effect of food neophobia on intention to eat raw and processed insects. The interaction between MSV and food neophobia was tested as well.

## 2. Materials and Methods

A convenience sample of 150 Italian subjects participated to the experiment on a voluntary basis. They were recruited through announcements on the university’s noticeboards and social media, which invited students to participate in a behavioral economics experiment, with no other information on the content and procedure. The data of two participants were dropped because of not completing the experimental procedure. The final sample, described in the following Table 1, consists of 148 subjects (106 females; *M*_age_ = 21.53, *SD*_age_ = 3.20).

**Figure 2:**
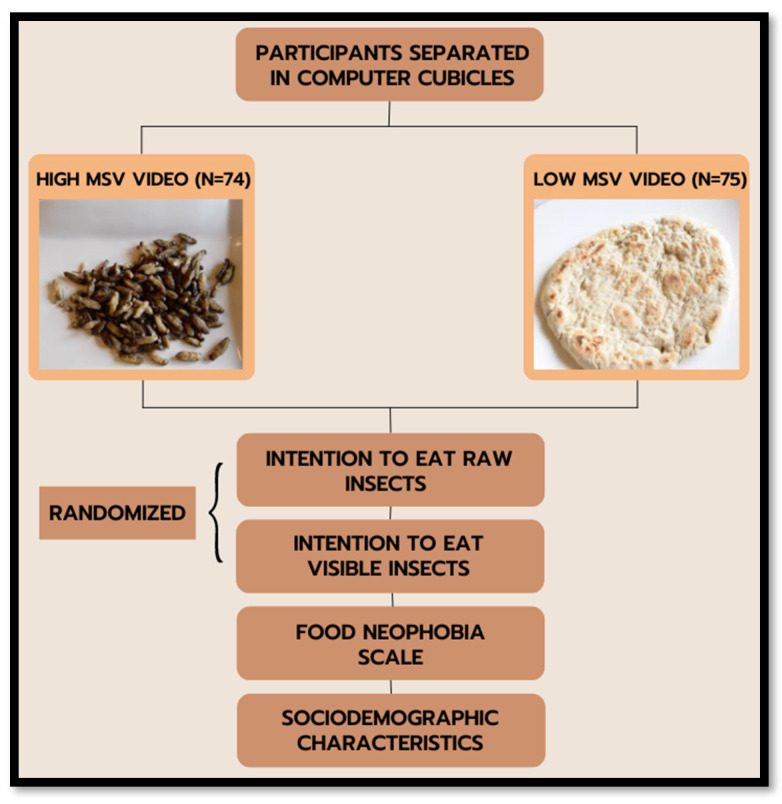
Flow of the experiment.

Only participants that declared that they have never eaten insect-based food were permitted to join the experiment. The experiment was completely computer-based. Participants met in a computer lab and each of them was identified with an ID number. Participants were assured of anonymity and informed that they were free to discontinue participation at any time without penalty. After giving their consent, participants were invited to watch a video on the computer screen. Specifically, each participant watched only one of the two different videos prepared for the experiment: one video showed the preparation and consumption of a tortilla made from cricket (*Acheta domesticus)* flour, whereas the other one showed the preparation and subsequent consumption of baked raw crickets. The experiment is double-blind, because participants were randomly assigned by the computer to one of the two experimental conditions, so that neither participants nor researchers were aware of who was receiving a particular treatment (see Figure 2 for an overview of the experiment).

Drawing on previous research on food with visible and non-visible insects [9,10,11,12,13,14,15], which show that the latter elicits less negative reactions compared to the former, we considered the video showing the preparation and consumption of baked raw crickets as a communication with higher MSV compared to the tortilla video. The two videos shared the main elements (e.g., the same person eating, the same environment for preparation) and structure, both being divided into two different moments: food preparation phase, lasting 1 min, and consumption phase, lasting 30 s. Participants were randomly assigned to one of the two experimental conditions (low MSV vs. high MSV). After watching the video corresponding to the experimental condition they had been assigned, participants completed several measures concerning neophobia, intention to eat raw insects, and intention to eat foods with non-visible insects (see Table 2). Finally, participants were thanked and debriefed. The entire experimental procedure had an approximate duration of 30 min and was supervised by two research assistants. The study was conducted according to the guidelines of the Declaration of Helsinki, and the ethical guidelines provided by the American Psychological Association. The research protocol was approved by the Review Board of the Psychological and Social Research Lab R. Gentile, Federico II University of Napoli, research protocol number 0152017.

### 2.1. Measures

**Food Neophobia Scale** (**FNS**)—The FNS by Pliner and Hobden [44] was administered. It consists of ten items rated on a 7-point scale (from disagree to agree). Tested for reliability, it proved excellent (α = 0.87). A single average score was calculated, with higher values indicating higher food neophobia.

**Intention to eat raw insects** (**INT-RAW**)—Participants’ intention to eat raw insects was measured using two pictures of raw (not processed—visible) cooked insects [49]. After viewing each picture, participants were asked how likely it was that they were willing to add the displayed food to their diet, if available on the market. Answers were collected on scales ranging from 1 (not at all likely) to 7 (totally likely). Items were averaged in a single score (Spearman–Brown ρ = 0.86).

**Intention to eat non-visible insects** (**INT-PRO**)—Participants’ intention to eat food with non-visible insects was measured using two images of processed foods containing non-visible insects [49]. After viewing each picture, participants were asked how likely it was that they were willing to add the displayed food to their diet, if available on the market (1—not at all likely, to 7—totally likely). A single average score was calculated (Spearman–Brown ρ = 0.93).

### 2.2. Statistical Analyses

Cronbach’s alpha was used to test the reliability of multi-item measures. According to common rules of thumb, acceptable values range from 0.70 to 0.90. This notwithstanding, Cronbach’s alpha has been shown to be very sensitive to the number of items included in the measure tested; for brief scales, values above 0.60 are also reasonable, because Cronbach’s alpha tends to underestimate the internal consistency of brief scales [37]. We used Spearman–Brown’s coefficient for two-item measures to obtain more accurate results [38]. In order to assess the significance of main and interactive effects, analysis of variance (ANOVA) was conducted by SPSS 27 (SPSS Inc., Chicago, IL, USA); partial η^2^ was adopted as effect size index throughout the manuscript. The accepted level of significance of the null hypothesis test was set at *p* < 0.05.

## 3. Results

**Figure 3:**
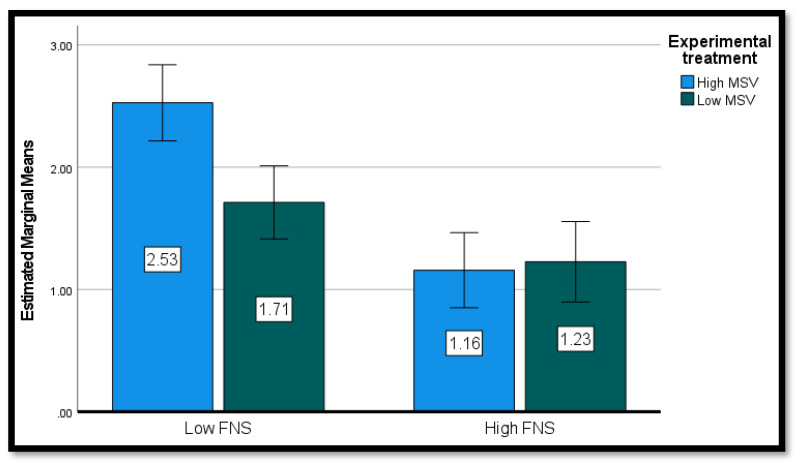
Conditional effects of FNS*MSV on the intention to eat raw insects.

To examine the effects of MSV, food neophobia, and their interaction on participants’ intention to introduce food with visible (INT-RAW) and non-visible insects (INT-PRO) into their diets, two ANOVAs were run with two between-participant factors, MSV (high versus low) and food neophobia (FNS—high versus low). In the first ANOVA, INT-RAW was the dependent variable. There was a significant effect of MSV on participants’ intention, which was more favorable in the case of high MSV message (M = 1.83, SD = 1.20) compared to the low MSV message (M = 1.49, SD = 0.95), *F* (1, 147) = 5.57, *p* = 0.020, partial η^2^ = 0.037. The effect of FNS was also significant: participants with low FNS showed higher INT-RAW (M = 2.10, SD = 1.30) compared to those with high FNS (M = 1.19, SD = 0.49), *F* (1, 147) = 34.52, *p* < 0.001, partial η^2^ = 0.193. Importantly, the crucial interaction between MSV and FNS proved significant, *F* (1, 147) = 7.84, *p* = 0.006, partial η^2^ = 0.052. Simple effects analysis (see Table 3 and Figure 3) showed that, in the low FNS condition, there was a significant difference between the intention of participants who were exposed to the high MSV message and those who saw the low MSV video, *t* (75) = 2.88, *p* = 0.005. In the high FNS condition, instead, no significant difference was found, *t* < 1.

**Figure 4:**
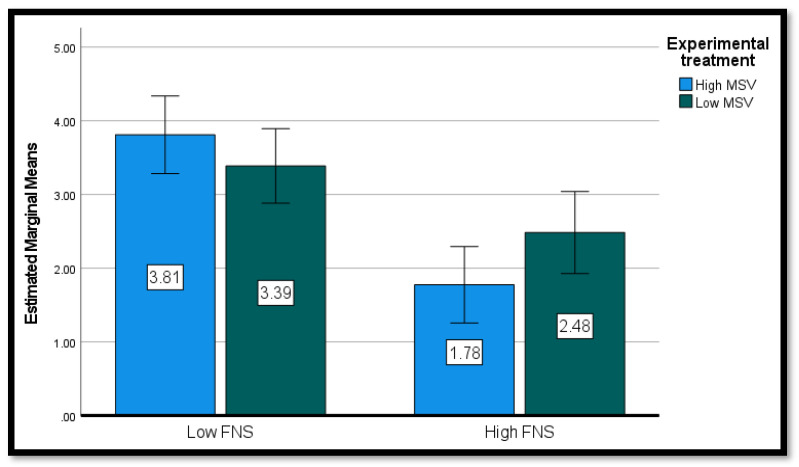
Conditional effects of FNS*MSV on the intention to eat processed insects.

The intention to eat foods with processed, non-visible insects (INT-PRO) was the dependent variable in the second ANOVA. The effect of MSV on participants’ intention was not significant, *F* < 1, whereas the effect of FNS was significant: participants with low FNS showed higher intention to eat food with processed insects (M = 3.59, SD = 1.81) compared to those with high FNS (M = 2.11, SD = 1.41), *F* (1, 147) = 30.33, *p* < 0.001, partial η^2^ = 0.174. The interaction between MSV and FNS was also significant, *F* (1, 147) = 4.50, *p* = 0.036, partial η^2^ = 0.030. Simple effects analysis (see Table 4 and Figure 4) showed that, in the low FNS condition, there was no significant difference between the intention of participants in the two different groups, *t* (75) = 1.02, *p* = 0.309. On the contrary, in the high FNS condition, the intention of participants exposed to the high MSV message was lower than the intention of participants exposed the low MSV message, *t* (69) = 2.16, *p* = 0.034.

## 4. Discussion

The insect supply chain is progressively approaching the market as demonstrated by the considerable attention shown by stakeholders. Legal frameworks are increasingly defined, and insect producers and processors have set up production facilities for different product lines ranging from protein extracts, fat extracts, dehydrated whole insects, etc. [50]. The considerable number of requests for scientific opinions addressed to EFSA [51] on new insect products reflects the interest of stakeholders and how close to market potential products are. At the same time, intense research activity has significantly increased the degree of knowledge for the main socio-economic drivers of demand, consumers attitudes, and personality dispositions [9]. It therefore becomes crucial to also extend research efforts to the analysis of potential communication and marketing strategies and the way different consumer profiles react to them.

Previous studies on this line of research focused mainly on the content of the information message [18,52,53]. However, so far very little attention has been paid to the effect of the level of intensity of communication messages on affective and arousal responses.

The present research has moved in this direction, using the construct of message sensation value (MSV) in an experimental setting to assess the intention to try two different types of insect-based foods. In the first case, the insects were clearly visible during the food preparation, whereas in the second they were incorporated as protein meal. Our results show that MSV, food neophobia, and their interaction significantly affect the willingness to consume insect-based food products with or without visible insects. The intensity of the message (low vs. high) shows a significant main effect on intention only in the case of raw insect-based foods. However, in this case the effect size appears rather modest (0.037). The second treatment (low intensity message) is not associated per se with any significant effect on the intention.

The individual level of neophobia also influences the willingness to consume. For both product typologies, the presence of neophobic traits reduces the willingness to consume. This result was widely expected and confirms the scientific evidence that identify neophobia as one of the main barriers that jeopardize the acceptance of insect foods. Besides being highly significant, neophobia also shows a very large effect size (0.193 for raw insect and 0.174 for processed insect-based food).

From a marketing perspective, the most qualifying result of the present research is the interaction between the experimental treatment (low vs. high MSV) and neophobia. In the case of participants with a low level of neophobia, a high-intensity message produced a significant effect on the intention to eat insect food with visible insects. In contrast, no effect is observed on the intention to consume food containing non-visible insects. When, on the other hand, neophobia is low, the treatment loses statistical significance in the case of foods with visible insects while it is significant when measuring the intention to consume foods with non-visible insects.

Present results highlight the importance attached to identifying and segmenting consumers, and the potential of screenings using measures of food neophobia for tailoring communication. For example, our results suggest that, if the communication target is expected (or it has been found) to have low levels of FNS, a communication characterized by high MSV may be useful to foster consumers’ willingness to eat food containing visible insects. On the contrary, if the target is expected (or it has been found) to have high levels of FNS, a communication characterized by high MSV may be totally ineffective, whereas a low MSV communication could be at least useful to foster consumers’ willingness to eat food containing non-visible insects.

Present results highlight the importance attached to consumer segmentation via latent constructs in order to strengthen the communication effect, avoiding the “one-size-fits-all” approach [54]. A well-fitted message that meets consumers’ needs and characteristics may have the potential to induce a behavioral shift, especially taking into account that behavioral and intention variables represent the result of the consumers’ decision processes [55] and are, therefore, suitable to identify consumer niches where new foods gain popularity first [56,57].

The research presents some limitations that should be acknowledged. First, from a methodological point of view, the study was conducted involving only Italian consumers, more precisely young inhabitants of the Campania region, thereby raising concerns regarding the generalization of results. This is especially important when taking into account that food neophobia is a cultural-dependent construct. In order to overcome this limitation, extensions of the current study—with large, heterogeneous, and cross-cultural samples—are needed. Second, the present study relied on a comparison between two videos which were supposed to be characterized by a different level of MSV. The experiment’s results support this expectation. Nevertheless, future study in this field should deepen these results exploring the effects of multiple messages with a wide range of MSV, measuring the perceived MSV of each message proposed to the research participants. This would be extremely useful to improve our knowledge about the effects of specific quantitative variations in MSV, which is still a scarcely studied topic.

## 5. Conclusions

For the first time, the present study tested the effect of messages with different MSV on the intention to eat visible and non-visible insects. The difference in MSV was found to exert a significant effect on participants’ intentions. Furthermore, MSV and food neophobia was shown to interact significantly. As a practical implication and from a marketing perspective, these findings offer new consumer segmentation schemes, complementing those already found in the literature such as the food related lifestyle [58]. In addition, the use of MSV allows specific messages to be calibrated to target certain population clusters. To achieve further intriguing results, future studies may include additional relevant variables to shed light on other possible interactive effects of MSV upon intention. For instance, the inclusion of the role of disgust might result in a better segmentation of consumers according to their preferences towards edible insects. Finally, given that processed insects are more likely to reach a well-established presence on Western supermarkets shelves, future studies on communications regarding different processed insect preparations—with different MSV—could help to better understand how to properly convey the right message to different consumer segments.

## Figures and Tables

**Table 1:** Descriptive statistics of the sample.

Characteristics	Frequency	Sample (%)
Gender		
Male	43	28.9
Female	106	71.1
Age		
18–23	110	73.8
24–31	39	26.2
Education		
High school	81	54.4
Bachelor level	42	28.2
Graduate	26	14.4
Household income (per month)		
Less than EUR 1000	27	18.1
Between EUR 1001 and 2000	57	38.3
Between EUR 2001 and 3000	48	32.2
More than EUR 3001	17	11.4
Household size (components’ number)		
1	2	1.3
2	1	0.7
3	21	14.1
4	77	51.7
More than 4	48	32.2

**Table 2:** Descriptive statistics of the Food Neophobia Scale.

Food Neophobia Scale (Cronbach’s α = 0.87)	Mean (SD)
I don’t trust new foods	2.75 (1.73)
I’m very particular about the foods I will eat	4.14 (2.13)
If I don’t know what is in a food, I won’t try it	3.70 (1.97)
I will eat almost everything *	3.35 (2.13)
I’m afraid to eat things I have never had before	2.97 (1.97)
I’m constantly sampling new and different foods *	3.17 (1.74)
I like to try new ethnic restaurants *	3.24 (1.96)
I like foods from different countries *	2.52 (1.64)
At dinner parties, I will try a new food *	2.93 (1.62)
Ethnic food looks too weird to eat	2.53 (1.61)

* Reversed items.

**Table 3:** Conditional effects of FNS*MSV on the intention to eat raw insects.

	Low MSV	High MSV
Low FNS	1.71 ^a^ (1.09)	2.52 ^b^ (1.37)
High FNS	1.23 ^c^ (0.66)	1.16 ^c^ (0.31)

Note: Mean scores of the intention to eat raw insects are reported in cells (standard deviation in parentheses). Different letters, per row, indicate statistically significant differences at *p* < 0.05.

**Table 4:** Conditional effects of FNS*MSV on the intention to eat processed insects.

	Low MSV	High MSV
Low FNS	3.38 ^a^ (1.80)	3.81 ^a^ (1.81)
High FNS	2.48 ^b^ (1.66)	1.78 ^c^ (1.08)

Note: Mean scores of the intention to eat processed insects are reported in cells (standard deviation in parentheses). Different letters, per row, indicate statistically significant differences at *p* < 0.05.

## Data Availability

Data will be made available upon request to the contact author.
